# Different Effects of Cold Stimulation on Reflex and Non-Reflex Components of Poststroke Spastic Hypertonia

**DOI:** 10.3389/fneur.2017.00169

**Published:** 2017-04-28

**Authors:** Sheng Li, Henry Shin, Ping Zhou, Xiaoyan Li

**Affiliations:** ^1^Department of Physical Medicine and Rehabilitation, McGovern Medical School, University of Texas Health Science Center at Houston (UTHealth), Houston, TX, USA; ^2^TIRR Memorial Hermann Research Center, TIRR Memorial Hermann Hospital, Houston, TX, USA; ^3^Guangdong Work Injury Rehabilitation Center, Guangzhou, China

**Keywords:** spasticity, hypertonia, stroke, reflex, cold stimulation, fusimotor

## Abstract

**Objective:**

To use an established biomechanical approach to quantify reflex and non-reflex responses from spastic–paretic elbow flexors in response to controlled cold and heat stimulation.

**Methods:**

Thirteen spastic–hemiplegic stroke subjects were tested in the experiment. The spastic elbow joint was stretched into extension for 50° at two speeds (5°/s and 100°/s) in a customized apparatus. Thermal stimulation (HEAT at heat pain threshold, COLD at 0°C, or BASELINE at room temperature) was applied to the thenar eminence of the contralateral hand immediately prior to stretching for at least 30 s.

**Results:**

Total torque was greater at 100°/s than at 5°/s. Total torque was significantly increased after COLD, but not HEAT as compared to BASELINE. When normalized to total torque at baseline, HEAT decreased total torque by 6.3%, while COLD increased total torque by 11.0%. There was no significant difference in the reflex torque among three thermal conditions.

**Conclusion:**

The findings demonstrate differentiated effects of cold stimulation on the total resistance from spastic muscles. They provide objective evidence for anecdotal clinical observations of increased muscle spasticity by cold exposure.

## Introduction

Spasticity is one of a multitude of factors that cause hypertonia. Spasticity is clinically defined as a phenomenon of velocity-dependent increase in resistance to external stretch, i.e., exaggerated stretch reflex (1). Alteration in spasticity can be a sign or reflection of underlying pathology, e.g., increased spasticity can be caused by urinary tract infection. Severity of spasticity could also be affected by many other factors, such as posturing, anxiety, and weather (2). Neurologically stable spinal cord-injured patients may present, or may be perceived to have, different levels of spasticity in the winter time (3) or even with strong air conditioning (4). However, these results are mixed (2). There are also case reports which showed that spasticity was increased by cold weather in stroke patients (5, 6). This pattern of change usually requires adjustment in treatment plan or options, such as increasing dose of botulinum toxin in the winter time.

Cold exposure increases the severity of spasticity in the affected muscles and overall muscle tone of the whole body. This clinical observation of increased tone in response to acute cold challenge is part of common thermal defense responses, including cutaneous vasoconstriction and shivering and non-shivering thermogenesis. The overall increase in muscle tone before the onset of overt shivering is termed “thermal muscle tone” (7). It is related to activation of the fusimotor drive *via* the thermoregulatory reflex during cold exposure (8). In an animal study (8), the efferent fusimotor activity to the rat gastrocnemius muscle was found to increase when the trunk skin was cooled for as short as 30 s by a water-perfused jacket and returned to normal when the skin was rewarmed. The cold-induced fusimotor activation enhanced the afferent discharges of the stretched muscle. However, this activation was blocked by inhibition of neurons in the rostral medullary raphe. In other words, thermal muscle tone is centrally mediated.

Spastic hypertonia consists of reflex-related component, i.e., hyperreflexia and non-reflex-related component (9–12). The hyperreflexia-related spaticity accounts for the commonly observed phenomenon of velocity-dependent increase in resistance. It is indicative of hyperexcitability of spinal stretch reflex circuits as a result of imbalanced descending pathways secondary to disinhibition after stroke (12). On the other hand, a number of peripheral muscular changes can contribute to hypertonia (13, 14). These factors include muscle fiber changes and altered tendon compliance. These peripheral changes may be secondary to paresis. When a paralyzed muscle is maintained in a shortened joint position, for example, a flexed elbow joint position, the muscle is often able to adapt to this situation and becomes shortened. As a result, sarcomeres are lost, muscle fibers become stiffer, and mechanical resistance to stretch increases, i.e., hypertonia (15). Additionally, there is an accumulation of viscous intramuscular substances, such as hyaluronan (16). These changes in the mechanical properties of muscles may gradually lead to increased muscle stiffness (17). These components are not adequately differentiated in common clinical examinations (18) or even in quantitative assessments of muscle stiffness in the laboratory setting (19).

Cold-induced changes in the severity of spasticity are easily perceived by patients and are often assessed with clinical scales, such as modified Ashworth scale (MAS). However, it remains unknown which components of spasticity are influenced by cold exposure. Since cold-induced thermal muscle tone is centrally mediated, its effect on spastic muscles could be quantified by changes in spasticity in the affected muscle in responses to experimentally imposed cold challenge to the contralateral limb. In this way, the peripheral mechanisms in the spastic muscle are not altered by cold stimulation. To achieve this goal, we have developed a thermal-stimulation/biomechanical-quantification approach in this study. In particular, we used a thermal stimulator to provide a brief yet controlled heat/cold stimulation to the contralateral hand in stroke survivors, while an established biomechanical approach was used to quantify the response from the spastic–paretic elbow flexors (20–23). Resistance to slow stretching (e.g., 5°/s) is considered to reflect the passive and non-reflex property of spastic muscles. The difference in resistance between fast (e.g., 100°/s) and slow stretching thus represents the reflex component of spastic muscles. As such, this unique approach allowed us to examine the effects of cold exposure on different components of spasticity.

## Materials and Methods

Thirteen spastic–paretic hemiplegic stroke subjects (five females, eight males, aged 53–78 years, history of stroke: 68 ± 42 months) participated in the experiment. Subjects were recruited from the TIRR Memorial Hermann outpatient clinic. Inclusion criteria were those who: (1) had spastic hemiplegia secondary to a single ischemic or hemorrhage stroke; (2) had a history of at least 6 months poststroke; (3) had elbow flexor spasticity of the impaired side greater than 0, but less than 3 (rated by MAS) in order to have sufficient range of motion for stretching; (4) were able to understand and follow instructions related to the experiment; and (5) were able to give informed written consent. The exclusion criteria were those who: (1) had a history of multiple strokes or bilateral involvement and (2) had presence of contracture that would limit full elbow range of motion on the impaired side. Spasticity of elbow flexors was MAS of 1+ and 1 in this experiment. The experiment was approved by the UTHealth Committee for the Protection of Human Subjects. All subjects gave written informed consent prior to participation.

**Figure 1:**
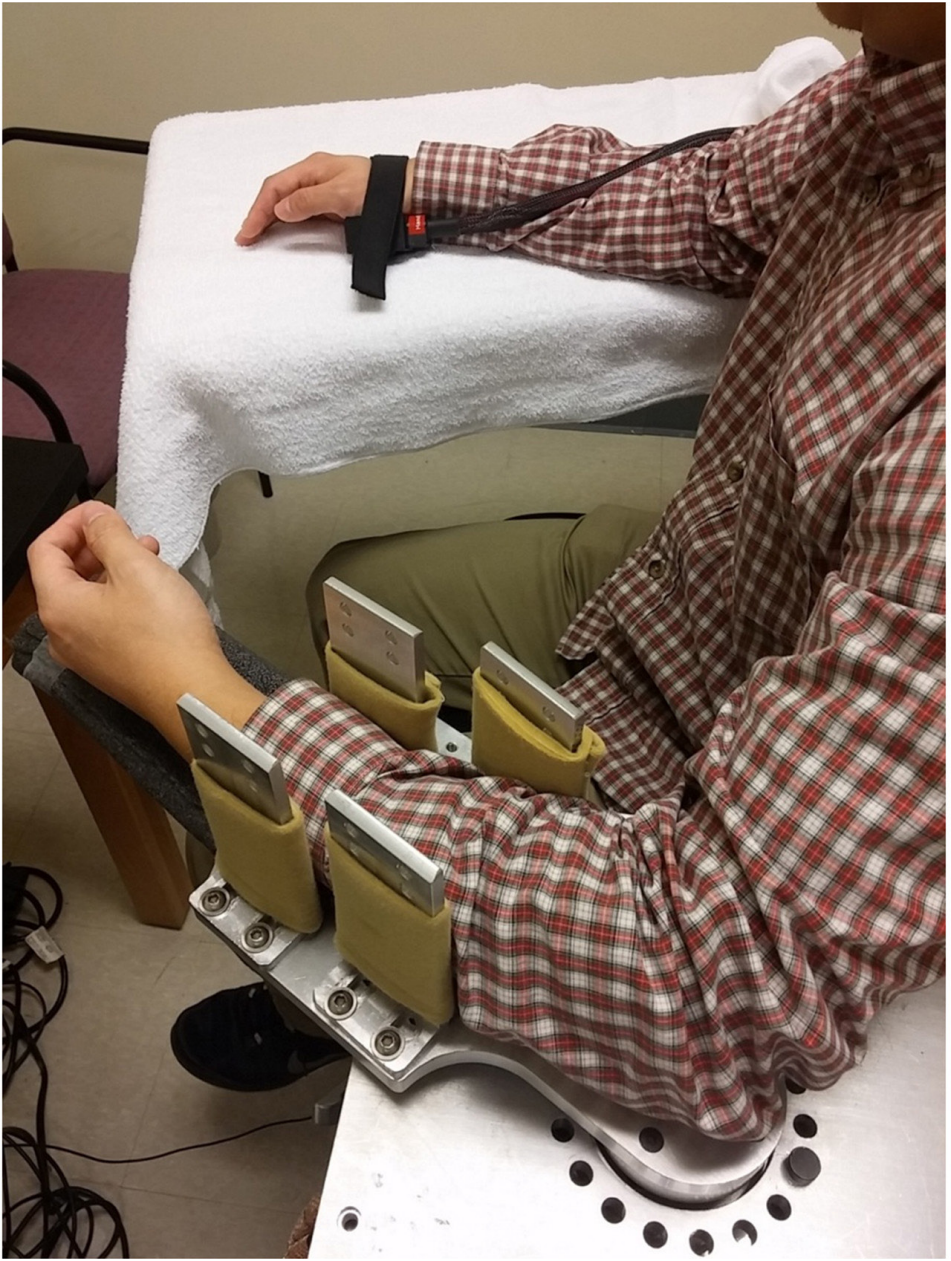
**Experimental setup**.

For this study, we adopted our recent experimental setup (23, 24). The subjects were seated on a height adjustable chair. The spastic limb was safely secured in a customized apparatus with the shoulder at approximately 45° of abduction and 30° of flexion. The center of the elbow joint and the axis of rotation of the servo motor were perfectly aligned to prevent translation and rotation of the arm. Four vertical plates were used to secure the forearm at the proximal and distal forearm (Figure 1). The subjects were instructed to keep the wrist, hand, and fingers relaxed during external stretching. The other arm of the subject rested symmetrically on another table.

### External Stretch

A similar stretching protocol was used (23). A ramp-hold stretching at two different speeds (5°/s and 100°/s) was applied. The range of stretching was 50° of elbow extension with reference to the resting joint angle. From the initial elbow joint position, a constant-velocity extension movement was imposed at the elbow until the elbow reached the predetermined end position. The elbow was then held in the end position for 2 s and returned to the initial position at the same velocity. A rest period of about 60 s was allowed between trials to allow adequate recovery and to minimize the influence of stretch history on the response to the subsequent stretch. Subjects were instructed explicitly not to support/facilitate or to oppose the external stretch, i.e., complete relaxation throughout the trials. Two velocities of 5°/s and 100°/s were used with three trials at each velocity.

### Thermal Stimulation

There were three thermal conditions: no thermal stimulation (BASELINE, i.e., room temperature), heat stimulation at heat pain threshold (HEAT), and cold stimulation at 0°C (COLD). A 45-s thermal stimulation was delivered to the skin of the contralateral hand *via* a thermode probe (the PATHWAY system, Medoc Ltd., Israel) immediately prior to stretching. As shown in Figure 1, a 30 mm × 30 mm advanced thermal stimulator (ATS) probe (thermode) was placed in the marked center of thenar eminence of the contralateral limb. The ATS delivers thermal stimuli at a temperature range of 0–52.5°C with a heating and cooling rate of up to 8°C/s. The thermode rested at room temperature (around 32°C) when not in use. During COLD, the temperature of the thermode decreased at 8°/s till 0°C (it takes about 4 s) and then remained at 0°C till the end of the 45-s trial. During HEAT, the temperature of the thermode increased from the baseline and was maintained at the predetermined heat pain threshold (around 43–45°C). During BASELINE, there was no change in the temperature of the thermode. For all conditions, the previously mentioned computerized external stretching was triggered at the end of the thermal stimulation. Each thermal condition was tested twice at each stretching speed. The interval between two thermal stimulation trials was 2 min. The order of stretching speed and thermal condition was randomized to avoid any bias caused by the stretching order.

### Data Analysis

Resistance torque was measured with a torque sensor (Model TRS 500, Transducers Techniques, CA, USA). An angular motion was recorded using encoder (HD FHA-25C-50-US250, Standard Incremental, 2,500 pulses per revolution). All signals were digitized at 1,000 samples/s on a PC with a BNC-2090A data acquisition board (National Instruments, Austin, TX, USA) using custom LabView software (National Instruments). Data were saved for offline analysis using a custom MATLAB (The MathWorks Inc.) program.

Angle and torque signals were analyzed to determine the biomechanical response of the stretch on the spastic elbow flexors (23). For each subject, peak torque was calculated for all speeds between the start and end of arm extension. Total torque was calculated by subtracting the baseline (pre-stretch) torque from the peak value. Reflex torque was defined as the difference in total torque between the fast (100°/s) and the slow (5°/s) stretching. To characterize the pattern of the response, average torque was calculated across all three trials for each speed and each thermal condition.

### Statistics

A two-way repeated measures ANOVA was applied to examine the main effects of stretching velocity (SPEED, two levels: 5°/s and 100°/s) and thermal stimulation (THERMAL, three levels: BASELINE, COLD, HEAT) on total torque. To compare changes in total torque from the baseline after thermal stimulation, a two-way ANOVA (SPEED and TEMP, two levels, hot/cold) was used. A One-way ANOVA (THERMAL) was used to investigate the impact of thermal stimulation on reflex torque. *p* < 0.05 was chosen to indicate statistically significant differences.

## Results

**Figure 2:**
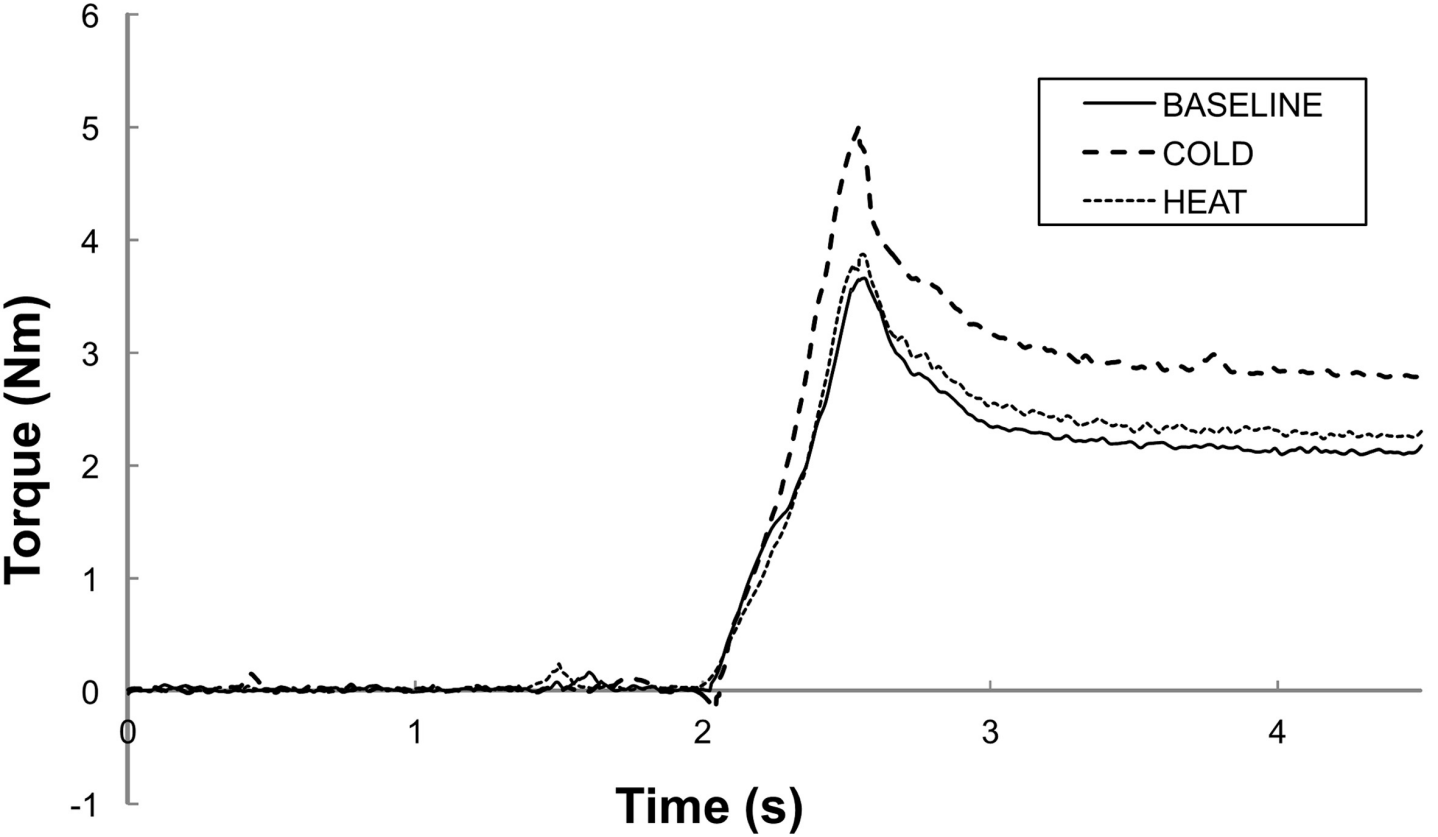
**Representative trials of total torque responses at a stretching speed of 100°/s**. BASELINE: room temperature, COLD: 0°C cutaneous stimulation to the contralateral thenar eminence; HEAT: thermal stimulation at the heat pain threshold to the contralateral thenar eminence. Note that the peak torque response is greater in COLD as compared to in HEAT and BASELINE. Both HEAT and BASELINE have similar torque peak torque responses.

**Figure 3:**
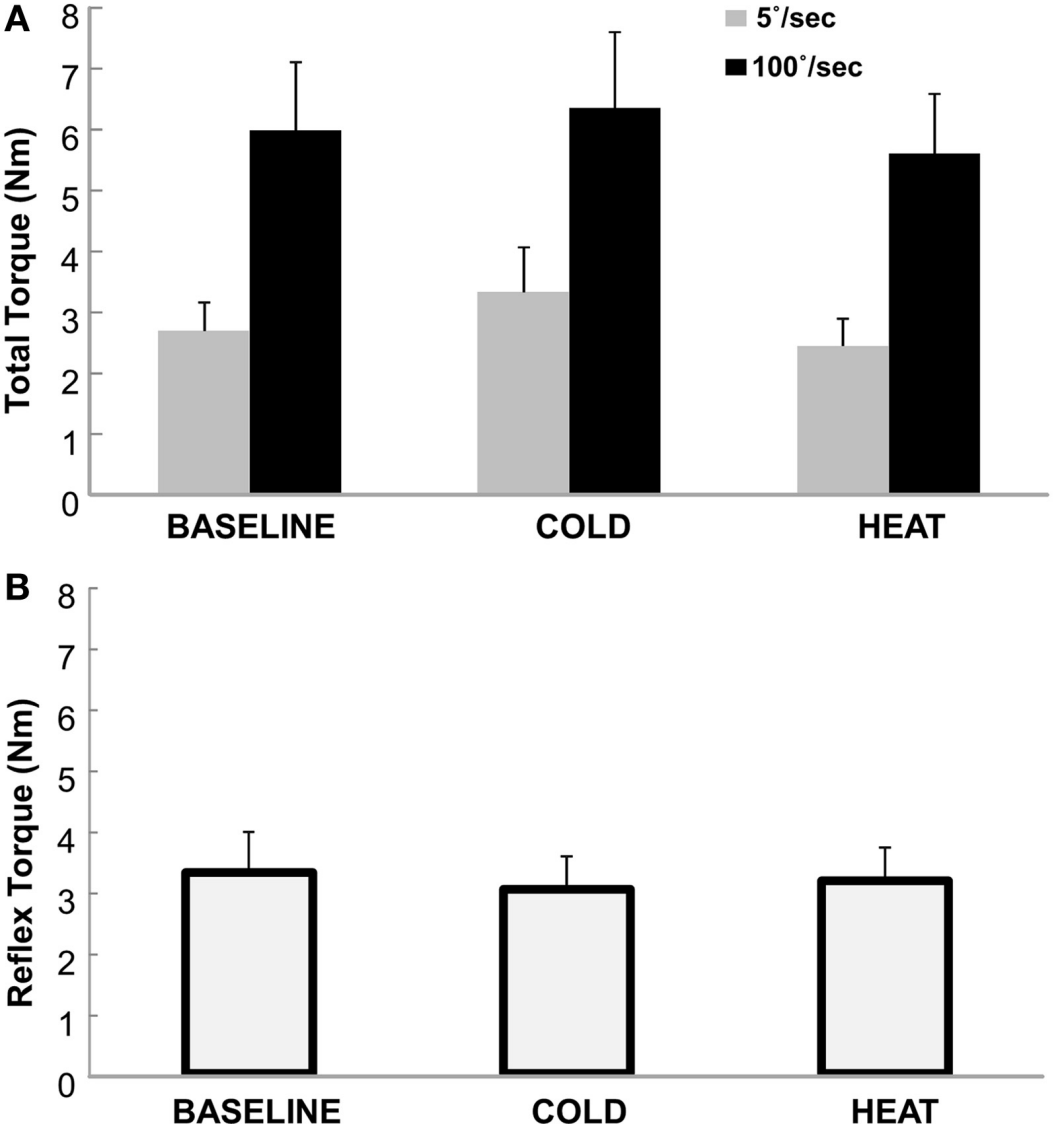
**(A)** Total Torque at BASELINE, COLD, and HEAT conditions. Gray bars indicate torque responses at slow stretch (passive component) and black bars indicate torque responses at fast stretch (passive + reflex component). A clear difference between speeds is visible, but not necessarily between temperatures. Mean and SEs are shown. **(B)** Reflex torque at BASELINE, COLD, and HEAT conditions. The reflex component of the total torque is the difference between the fast and slow torque responses (Total Torque at 100°/s − at 5°/s). Mean and SE are shown.

Total torque was significantly affected by both stretching speed and thermal stimulation (Figure 2). According to the two-way ANOVA analyses, there were main effects of SPEED [*F*(1,12) = 27.65, *p* < 0.0001] and THERMAL [*F*(2,24) = 3.88, *p* = 0.035]. No significant interactions were observed. Averaged across different thermal conditions, total torque was significantly greater at 100°/s (5.98 Nm) than at 5°/s (2.82 Nm). Averaged across different speeds, total torque was 4.33 Nm at BASELINE, 4.84 Nm during COLD, and 4.02 Nm during HEAT (Figure 3A).

**Figure 4:**
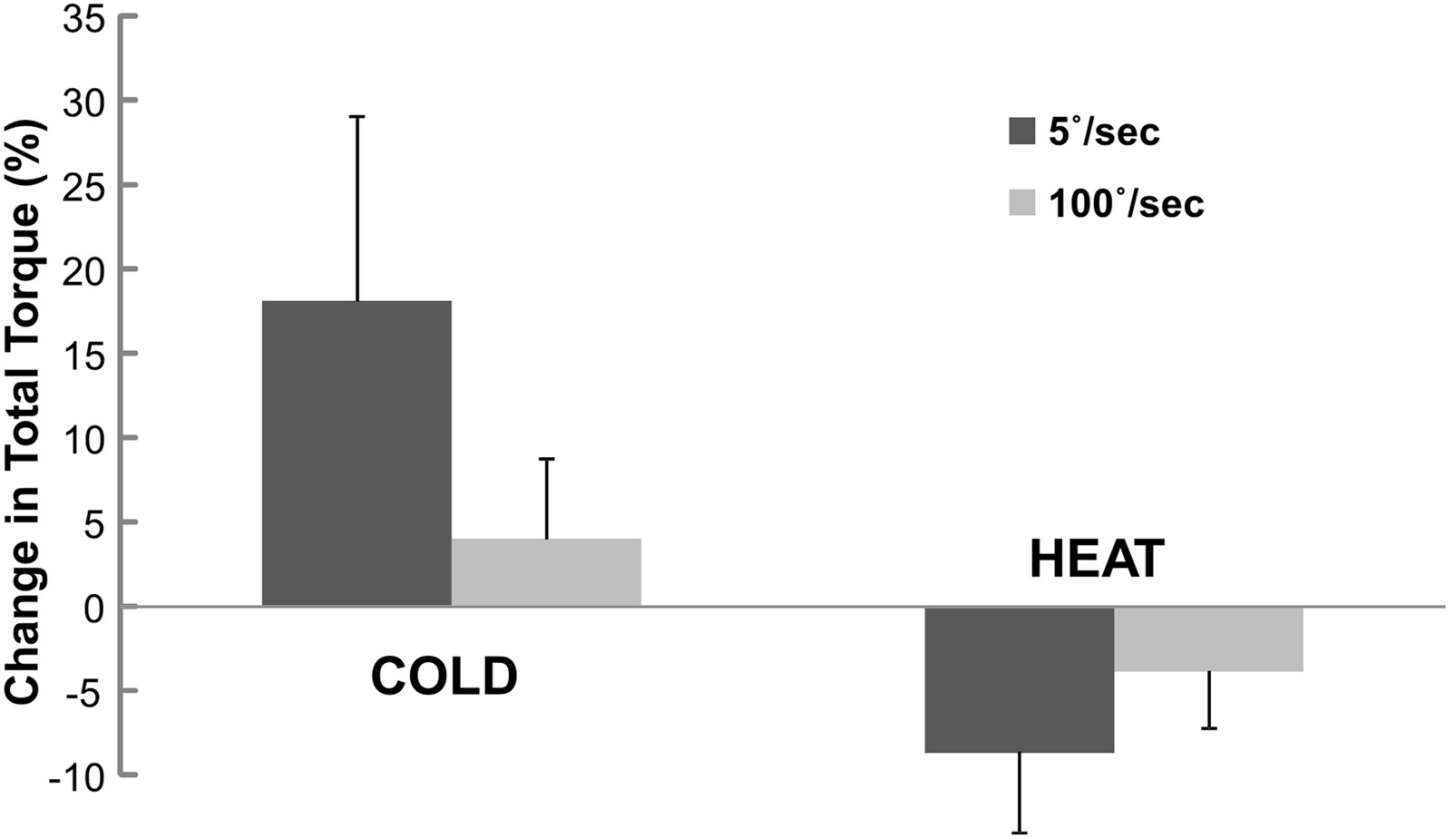
**Change in total torque at COLD and HEAT stimulation**. Change in total torque refers to percent difference in total torque between COLD/HEAT and BASELINE that is normalized to total torque at BASELINE. Positive change means increase in total torque. Mean and SEs are shown.

When normalized to the total torque at BASELINE, the percent change in total torque was also speed and temperature-dependent (Figure 4). Two-way ANOVA analyses showed main effects of SPEED [*F*(1,12) = 0.473 *p* = 0.505], TEMP [*F*(1,12) = 9.078 *p* = 0.011] and a significant interaction SPEED × TEMP [*F*(1, 12) = 6.907, *p* = 0.022]. Tukey *post hoc* tests further revealed that the percent torque change was significantly greater by COLD (18.1%) than HEAT (−8.7%) at slow speed, but there was no difference in torque change between COLD (4.0%) and HEAT (−3.9%) at fast speed of stretching. Overall, COLD increased total torque by 11.0%, while HEAT decreased total torque by 6.3%.

The reflex torque, obtained visually by subtracting the gray bars from the corresponding black bars on Figure 3A, however, did not differ significantly across three thermal conditions [*F*(1,12) = 0.661, *p* = 0.551]. They were 3.29, 3.01, and 3.15 Nm for BASELINE, COLD, and HEAT, respectively (Figure 3B).

## Discussion

In this experiment, a novel thermal-stimulation/biomechanical-quantification approach was used to quantify spastic hypertonia of elbow flexors in stroke subjects when thermal stimulation was applied to the contralateral hand in three thermal conditions: BASELINE, COLD, and HEAT. There were no changes in the biomechanical configuration or sensorimotor condition of the spastic limb during subsequent external stretches across different thermal conditions. The thermal-stimulation/biomechanical-quantification approach allows a unique opportunity to individually assess the reflex and non-reflex components of spastic hypertonia. In addition to confirming velocity-dependent increases in total torque, the novel findings included (1) total torque was significantly increased after COLD, but not HEAT as compared to BASELINE and (2) reflex torque did not differ significantly across the three thermal conditions. Our findings of no significant change in reflex torque across thermal conditions and increased total torque after cold stimulation suggest that cold stimulation specifically modulates the non-reflex component without affecting the reflex component in stroke survivors.

This study found that cold stimulation primarily alters non-reflex, passive stiffness of spastic muscles in stroke subjects. It is important to point out that many factors could contribute to non-reflex, passive stiffness, such as viscoelasticity of the muscle (14, 25), changes in muscle property (26), and accumulation of extracellular deposits (27). Unfortunately, we are not able to differentiate these factors in this study. Our finding was consistent with reports from an earlier study (7). A brief cold exposure specifically increased overall muscle tone, but did not alter fine neuromotor control during force production. The increase in the non-reflex response and the overall muscle tone during cold exposure suggests a central mechanism. This cold-induced increase in overall muscle tone is likely part of cold-defense mechanisms *via* activation of neurons in the rostral medullary raphe (8). In contrast, no effect of cold stimulation on the reflex component suggests that the excitability of reflex pathways is not affected.

The finding of no significant effect of cold stimulation on the reflex component is not trivial. The stretch reflex is a monosynaptic reflex that provides automatic regulation of skeletal muscle. Skeletal muscle contains extrafusal and intrafusal muscle fibers. Extrafusal fibers are innervated by alpha motor neurons, mediating muscle contraction. Intrafusal fibers constitute the muscle spindle. These intrafusal fibers are innervated by gamma motor neurons. The muscle spindle serves as mechanoreceptors for the muscle and provides feedback to the central nervous system. When a muscle is lengthened by passive stretch, both the change in muscle length and its rate are detected by muscle spindles (primarily group Ia and II afferent fibers). Golgi tendon organs also send information on muscle tension through group Ib afferent fibers. The increase in neuronal activity in these fibers in turn increases alpha motor neuron activity in the stretched muscles through the stretch reflex. This causes the extrafusal muscle fibers to contract and thus resist the muscle lengthening or stretching. Meanwhile, Ia afferent fibers also synapse with Ia inhibitory interneurons, producing relaxation of the antagonist muscles (“reciprocal inhibition”). In summary, gamma motor neurons regulate muscle spindles (fusimotor system) and thus the reflex sensitivity.

By subtracting the torque response at 5°/s from that at 100°/s, reflex torque reflects the aggravated reflex response to a fast external stretch. This velocity-dependent reflex torque is likely mediated by velocity-sensitive spindle afferent inputs (Ia and II spindle afferents) (22, 28) and hyperexcitable spinal motor neurons (9, 12, 29). It is viewed as a result of unopposed descending inputs to spinal motor neuron pools from hyperexcitable medial reticulospinal pathways (12, 30). Cold stimulation is known to relate to the activation of the fusimotor drive *via* the thermoregulatory reflex (8). The cold-induced fusimotor activation enhanced the afferent fusimotor discharges. No cold effect on the reflex torque suggests that the cold-induced fusimotor activation does not increase stretch reflex responses in spastic flexors. These findings are in general in consistence with previous findings (31, 32). These studies reported that the reflex amplitude was not disproportionally affected by changes in fusimotor function—by an after-effect of voluntary contraction, cutaneous mechanical stimulation, or electrical nerve stimulation. However, our findings extended previous reports that the fusimotor function could significantly alter the non-reflex component of spastic hypertonia. Further research needs to investigate how the fusimotor activation changes the non-reflex component of passive resistance in spastic muscles.

Limitations of this study include the number of stretch measurements made at each thermal condition. The muscle reflex response is highly variable, but also typically shows a decrease in amplitude with each successive stretch within a short period of time. Only two measurements were made in each thermal condition as to not heavily weigh whichever condition was measured first. This potential bias was also remediated by randomizing the order of the thermal condition. Thermal stimulation was applied only to the contralateral side in this study. The effect of thermal stimulation would be more significant if it was applied on the same side or directly on the target muscles. However, the potential confounding factor is that increased passive stiffness could be induced directly by cold stimulation. In future studies, comparisons of the effects of thermal stimulation to the spastic muscles and on the contralateral side may be able to provide useful information.

## Conclusion

The findings provide objective evidence for anecdotal clinical observations of increased muscle spasticity due to cold exposure. Increased spastic hypertonia appears to be primarily caused by an alteration in the non-reflex component of spastic hypertonia at a central level.

## Ethics Statement

The experiment was approved by a local Institutional Review Board.

## Author Contributions

SL, HS, PZ, and XL: study concept and design; analysis and interpretation; critical revision of the manuscript for important intellectual content. HS and XL: acquisition of data. SL: study supervision.

## Conflict of Interest Statement

The authors declare that the research was conducted in the absence of any commercial or financial relationships that could be construed as a potential conflict of interest.
